# Prevalence and Clinical Implications of Hemosiderin Deposits in Recent Small Subcortical Infarcts

**DOI:** 10.1212/WNL.0000000000209973

**Published:** 2024-10-24

**Authors:** Yu-Yuan Xu, Francesca M. Chappell, Maria Del C. Valdés Hernández, Carmen Arteaga-Reyes, Una Clancy, Daniela Jaime Garcia, Stewart Wiseman, Michael S. Stringer, Michael Thrippleton, Yajun Cheng, Junfang Zhang, Xiaodi Liu, Angela C.C. Jochems, Fergus Doubal, Joanna M. Wardlaw, for the MSS3 Study Group

**Affiliations:** From the China National Clinical Research Center for Neurological Diseases (Y.-Y.X.), Beijing Tiantan Hospital, Capital Medical University; Centre for Clinical Brain Sciences (F.M.C., M.D.C.V.H., C.A.-R., U.C., D.J.G., S.W., M.S.S., M.T., A.C.C.J., F.D., J.M.W.), UK Dementia Research Institute, University of Edinburgh, United Kingdom; Department of Neurology (Y.C.), West China Hospital, Sichuan University, Chengdu; Department of Neurology & Institute of Neurology (J.Z.), Ruijin Hospital affiliated with Shanghai Jiao Tong University School of Medicine; and Division of Neurology (X.L.), Department of Medicine, LKS Faculty of Medicine, The University of Hong Kong, China.

## Abstract

**Background and Objectives:**

A quarter of ischemic strokes are of lacunar clinical subtype and have an underlying recent small subcortical infarct (RSSI), but their long-term outcomes remain poorly characterized. Hemosiderin deposits (HDs) have been noted in RSSIs at chronic stages and might mimic primary hemorrhage. We characterized HDs' morphology, frequency, and clinical relevance.

**Methods:**

Participants with RSSIs were identified from a prospective longitudinal study and evaluated on 3T MRI including susceptibility-weighted imaging (SWI) from stroke diagnosis to 12 months. We categorized HDs in RSSIs on SWI at all available time points into 4 types (spots, smudge, rim, cluster) and assessed their associations with demographic factors, stroke-related factors, and image markers with adjusted logistic regression.

**Results:**

HDs were observed in 43 (55.0%) of 108 participants within 3 months and 83 (76.9%) of 108 within 12 months after stroke onset. The mean time to first detection of HDs was 87 (interquartile range 53–164) days. A “rim” pattern (similar to late appearance of primary hemorrhage) occurred in at least 26.5% of RSSIs at all follow-up time points, mainly those located in the lentiform/internal capsule (50.0%) or thalamus (36.4%). Infarct volume (odds ratio [OR] 1.003, 95% CI 1.001–1.006; *p* = 0.004) and the total small vessel disease (SVD) score at baseline (OR 2.50, 95% CI 1.28–4.86, *p* = 0.007) independently predicted HDs at 12 months. HDs were positively associated with more lacunes (OR 1.60, 95% CI 1.13–2.26, *p* < 0.01), but not the Fazekas score, number of microbleeds, basal ganglia mineral deposit score, or clinical outcomes.

**Discussion:**

HDs occur commonly in RSSIs and may be associated with infarct volume and SVD score. Hemosiderin “rim” is common in RSSIs, urging caution to avoid mistaking ischemic RSSI for primary hemorrhage in subacute and chronic stages.

## Introduction

Recent small subcortical infarcts (RSSIs), also known as lacunar ischemic strokes, account for approximately 25% of ischemic strokes.^1-3^ The long-term evolution of RSSIs is not fully understood. Previous studies suggest that RSSIs could undergo various long-term transformations seen on brain imaging with MRI or CT scanning, including partial or complete cavitation (lacune formation), remaining hyperintense on MRI or hypointense on CT, thus resembling a white matter hyperintensity (WMH), or even disappearing.^4-6^

Recently, with increasing use of 3T MRI scanners and blood-sensitive sequences such as susceptibility-weighted imaging (SWI), new features indicating hemosiderin deposition have been reported during the long-term evolution of RSSIs. These features appear as “rim” or “smudge” patterns.^3^ One study identified hemosiderin foci in 10% of RSSIs during long-term follow-up MRI (mean time 33.5 months), suggesting it might represent hemorrhagic transformation.^7^ Another study found that hemorrhagic foci occurred in 44.6% of RSSIs on SWI at approximately 12 months (median 449 days) after stroke.^8^ We noted similar hemosiderin deposits (HDs) in RSSIs during follow-up in a longitudinal study of participants with lacunar ischemic stroke, the Mild Stroke Study 3 (MSS3).^9^ However, the morphology, prevalence, and underlying mechanisms of HDs remain incompletely understood and warrant further exploration.

Because some HDs can resemble a rim as seen in end-stage small subcortical hemorrhages, it is important to investigate the frequency and factors associated with HDs to reduce potential confusion of lacunar ischemic stroke with small previous hemorrhage in clinical practice. Hence, we aimed to characterize the appearance and evolution of HDs in RSSI, to determine the morphology, frequency, and stage of hemosiderin appearance and identify potential predictors, risk factors, and associations.

## Methods

### Study Participants

We identified participants from the prospective observational MSS3 that started recruitment in August 2018 and completed 1-year follow-up in October 2021 (ISRCTN 12113543). The MSS3 study protocol is published.^9^ In brief, MSS3 prospectively recruited participants with ischemic stroke who presented to the Lothian Stroke Service in Edinburgh and surrounding areas, United Kingdom, with symptoms of mild stroke (i.e., NIH Stroke Scale [NIHSS] score ≤7 and modified Rankin Scale [mRS] score ≤2) due to either a lacunar or cortical stroke subtype. All participants were evaluated for cardiac and carotid sources of embolism, with fewer than 10% of those with lacunar strokes found to have either. All participants had a thorough clinical examination by dedicated stroke physicians and detailed diagnostic workup including diagnostic brain MRI or CT at presentation.^9^ Study 3T brain MRI scans were then obtained at baseline (1–3 months after stroke) and within 6-month and 12-month visits to assess changes in vascular brain lesions.

For this analysis, we considered all MSS3 study participants with clinical lacunar syndrome and a RSSI on MRI. We excluded study participants (N = 122) with cortical syndrome/infarct (N = 99), obvious early (i.e., within the first few days of stroke) hemorrhagic transformation (N = 2), missed scanning at 6-month or 6–12 month follow-ups (N = 9), poor image quality caused by motion artifact (N = 1), and other reasons (N = 11) (eFigure 1).

All participants received usual guideline stroke prevention therapies, including antiplatelet therapy or anticoagulation for those with atrial fibrillation, as well as antihypertensive and lipid-lowering treatments. Thrombolysis with guideline-based alteplase was administered in the acute phase when appropriate.

### Standard Protocol Approvals, Registrations, and Patient Consents

The study was approved by the Southeast Scotland Regional Ethics Committee (18/SS/0044). Informed consent was obtained from all participants in the study. All procedures performed in studies involving human participants were in accordance with the ethical standards of the institutional and/or national research committee and with the 1964 Helsinki Declaration and its later amendments or comparable ethical standards.

### Data Collection and Management

Patient demographics, vascular risk factors (i.e., hypertension, diabetes, hyperlipidemia, smoking status, and history of stroke or transient ischemic attack [TIA]), and medications were collected systematically at each follow-up visit. The NIHSS score at baseline and the mRS score and Montreal Cognitive Assessment (MoCA) test score at 12-month follow-up were also collected. Therapy was categorized as antiplatelet therapy or anticoagulation prescribed at the date of the baseline visit, within 3 months of stroke onset. Smoking status was classified as never smoked or ever smoker (current or ex-smokers). The poor functional outcome at 12-month follow-up was defined as an mRS score ≥2, and the cognitive outcome at 12-month follow-up was assessed using the MoCA.

### MRI Acquisition and Analysis

All participants underwent research MRI on a 3T Siemens PRISMA scanner with a 32-channel head coil (Siemens Healthcare, Erlangen, Germany) throughout to acquire sagittal 3D fluid-attenuated inversion recovery images, axial 3D T2-weighted images, sagittal 3D T1-weighted images, and axial 3D SWI sequences. 3D SWI acquisition parameters were as follows: TR = 28 milliseconds; TE = 20 milliseconds; acquired voxel size = 0.63 × 0.63 × 3 mm. Full details of the brain imaging scanning protocol are published.^9^

The assessment of RSSIs involved more than 5 raters who were blinded to clinical details. Old infarcts and all small vessel disease (SVD) features were recorded, as defined by the STRIVE criteria.^2,9^ These features included lacunes, white matter hyperintensities, Fazekas scores, cerebral microbleeds, perivascular spaces, iron deposits in the basal ganglia, and the total SVD score.^10^ All HDs on SWI were assessed by Y.-Y.X., blinded to the participants' clinical details. All the image assessment was checked by a senior neuroradiologist (J.M.W.).

When assessing the features of HDs in RSSIs, only the index infarcts were analyzed at each follow-up MR scanning. The index infarct refers to the acute infarct causing the primary stroke symptoms and physical signs consistent with a clinical stroke syndrome leading to initial presentation to stroke services. All follow-up scans were thoroughly reviewed. HDs observed at any visit within the first 6 months were recorded as positive at the 6-month follow-up. Similarly, all HDs observed from the 6-month visit to 12-month visit were recorded as positive at the 12-month follow-up. The time duration from the index RSSI onset to the follow-up imaging capturing the initial appearance of HDs was recorded.

**Figure 1:**
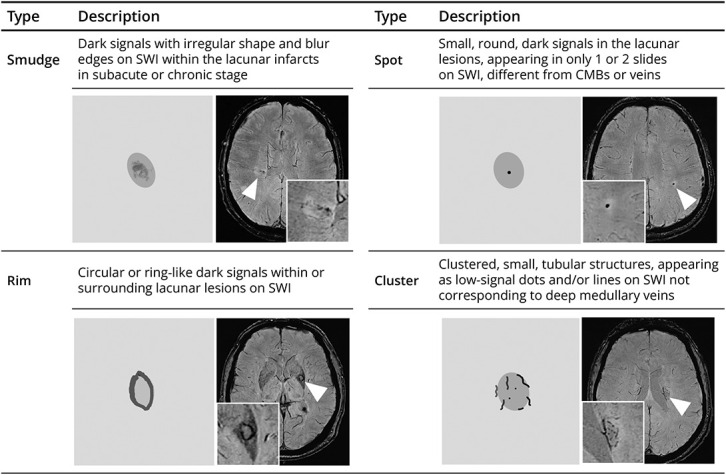
Morphologies and Description of Hemosiderin Deposits CMB = cerebral microbleed; SWI = susceptibility-weighted imaging.

Using SWI, we categorized HDs in SSI into 4 types, spots, smudge, rim, and cluster (Figure 1), with none as a fifth type. The location of RSSI was categorized as anterior circulation (blood supply from internal carotid arteries) and posterior circulation (blood supply from vertebral arteries) regions and further divided into 4 regions to examine the HD distribution: basal ganglia region (internal capsule, caudate, and putamen), thalamus, white matter region (centrum semiovale, including subcortical and periventricular, optic radiation, and corpus callosum), and subtentorial region (brain stem and cerebellum).^9^ The volume of index RSSIs was calculated according to the following formula: 0.5 × the maximum R-L axial width × the maximum A-P axial width × the vertical height (mm^3^).^11^

### Statistical Analysis

Data analyses were conducted using SPSS 22 (IBM SPSS Inc, Chicago, IL). Categorical variables were reported as absolute numbers with percentages, and continuous variables were reported as mean and standard deviation (SD) or median with the interquartile range (IQR). Categorical variables were compared by the χ^2^ test while the *t* test (normally distributed variables) or Mann-Whitney *U* test (non-normally distributed data) was used to compare continuous variables between the HD and non-HD groups.

The binary logistic regression model was used to investigate the associations between RSSI location/volume and HD presence at the 12-month follow-up. Adjustment factors included age, sex, NIHSS, vascular risk factors, and therapy (antiplatelet or anticoagulant), chosen for their clinically plausible relationship with HDs. Because the total SVD score was significantly correlated with individual SVD features, they were incorporated separately into the prediction models (model 1 and model 2). The collinearity of individual SVD features was also tested. The receiver operating characteristic curve was used to evaluate the fit of the logistic regression models.^12^

Moreover, 2 separate analyses were conducted: one examining the relationship between HDs and poor functional outcomes (defined as mRS score ≥2) using binary logistic regression and another assessing the association between cognitive outcomes (measured by MoCA) using multiple linear regression. Cases with missing data for mRS or MoCA were excluded. To ensure robust estimates, we dichotomized the mRS score into 2 groups: <2 and ≥2. The covariates included age, sex, NIHSS, vascular risk factors, and total SVD scores.

A quantile-quantile plot and a histogram of residuals were used to verify the normality assumptions of the linear regression model (eFigure 2). In addition, to evaluate the agreement between predicted and observed values, we used calibration plots and statistical metrics for 3 logistic regression models (eFigure 3) with R 3.4.2, rms package.^13,14^

### Data Availability

Supporting data of this study are available from the corresponding author on reasonable request.

## Results

### Participant Characteristics at Recruitment

108 of 230 participants with stroke recruited to the MSS3 (mean age 63.76 ± 11.45 years, 68.5% male, median NIHSS 1.57 ± 1.38) with symptomatic RSSIs meeting the inclusion criteria were included in this analysis (eFigure 1). The main reason for exclusion was having a cortical stroke.

Among the 108 with RSSIs, 83 (76.9%) developed HDs at some point during the 12-month study period. Table 1 presents the demographic, clinical, and imaging characteristics based on the presence or absence of HDs. Participants with HDs were less likely to have diabetes (19.3% vs 44.0%, *p* = 0.01). There was no difference in history of stroke or TIA, hypertension, hypercholesterolemia, and smoking between presence and absence of HDs. Only 2 of 108 participants received alteplase treatment in the acute phase, both of whom developed HDs (Table 1). Participants received either single antiplatelet therapy (aspirin or clopidogrel, n = 98, 90.7%) or anticoagulant therapy (n = 10, 9.3%) after the stroke onset, with no difference between HD and non-HD groups (antiplatelet 84.0% vs 92.8%, anticoagulant 16.0% vs 7.2%, *p* = 0.19) (Table 1).

**Table 1:** Baseline Characteristics

	Total (N = 108)	Non-HDs (N = 25, 23.1%)	HDs (N = 83, 76.9%)	*p* Value
Age, y, mean ± SD	63.76 ± 11.45	65.81 ± 9.39	63.15 ± 11.99	0.31
Male sex, n (%)	74 (68.5)	16 (64.0)	58 (69.9)	0.58
BMI, kg/m^2^, mean ± SD	28.29 ± 4.92	29.40 ± 4.79	27.95 ± 4.93	0.14
NIHSS score, median (IQR)	1 (0–2)	1 (1–2)	1 (0–2)	0.99
History				
Stroke or TIA, n (%)	20 (18.5)	7 (28.0)	13 (15.7)	0.16
Diabetes, n (%)	27 (25.0)	11 (44.0)	16 (19.3)	0.01
Hypercholesterolemia, n (%)	84 (77.8)	20 (80.0)	64 (77.1)	0.76
Hypertension, n (%)	81 (75.0)	21 (84.0)	60 (72.3)	0.24
Smoking, n (%)	57 (54.3)	11 (44.0)	46 (57.5)	0.24
Thrombolysis, n (%)	2 (1.9)	0	2 (1.7)	0.43
Second prevention therapy				
Anticoagulant, n (%)	10 (9.3)	4 (16.0)	6 (7.2)	0.19
Antiplatelet, n (%)	98 (90.7)	21 (84.0)	77 (92.8)	
Image features				
Total SVD score, median (IQR)	2 (1–3)	2 (1–2)	2 (1–3)	0.04
No. of microbleeds, median (IQR)	0 (0–1)	0 (0–0)	0 (0–1)	0.11
No. of lacunes, median (IQR)	2 (0–5)	0 (0–2)	2 (0–5)	0.01
Basal ganglia iron deposit score, median (IQR)	2 (1–3)	2 (1–2)	2 (1–3)	0.72
Fazekas score, median (IQR)	3 (2–5)	3 (2–4)	3 (2–5)	0.69
RSSI volume, mm^3^, mean ± SD	736.3 ± 1325.6	171.2 ± 277.2	906.6 ± 1464.1	<0.001
WMH volume, mL, mean ± SD	16.64 ± 18.45	19.54 ± 24.14	15.76 ± 16.43	0.74
Location category				
Anterior circulation, n (%)	66 (61.1)	9 (36.0)	57 (68.7)	0.03
Posterior circulation, n (%)	42 (38.9)	16 (64.0)	26 (31.3)	
Clinical outcome				
mRS score ≥2, n (%)	21 (19.4)	7 (29.2)	14 (16.9)	0.18
MoCA score, median (IQR)	27 (25–29)	27 (24–28)	27 (25.5–29)	0.92

Abbreviations: BMI = body mass index; MoCA = Montreal Cognitive Assessment; mRS = modified Rankin Scale; NIHSS = NIH Stroke Scale; SVD = small vessel disease; TIA = transient ischemic attack; WMH = white matter hyperintensity.

Compared with those without HDs, participants with HDs had a higher total SVD score at baseline (2.33 ± 1.28 vs 1.72 ± 1.28, *p* = 0.04) and more lacunes (3.40 ± 3.94 vs 1.48 ± 2.62, *p* = 0.006). The index RSSIs that developed HDs were more likely to be large in volume (906.6 ± 1464.1 mm^3^ vs 171.2 ± 277.2 mm^3^, *p* < 0.001) and located in the anterior circulation region (68.7% vs 36.0%, *p* = 0.03). There were no significant differences in the number of microbleeds (0 [IQR 0–0] vs 0 [IQR 0–1], *p* = 0.11) or in WMH volume (19.54 ± 24.14 vs 15.76 ± 16.43, *p* = 0.74) between the groups with and without HDs (Table 1).

### Categories and Evolution of HDs

**Figure 2:**
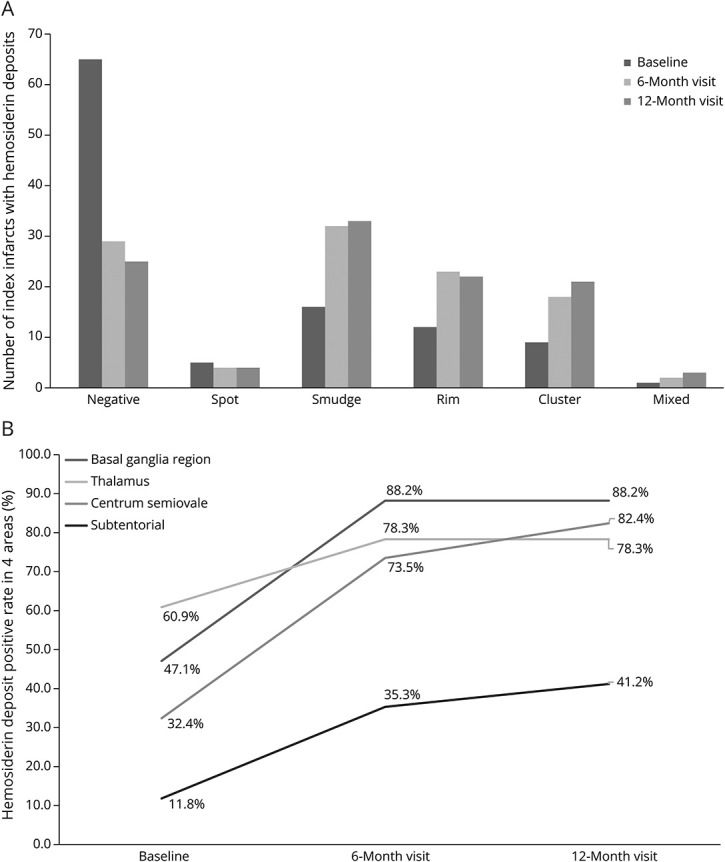
Categories and Evolution of HDs Figure 2A illustrates the quantity change of different HD patterns at the baseline, 6-month, and 12-month follow-ups. Figure 2B shows the increased positive rates of HDs in 4 areas at 3 time-point visits. HD = hemosiderin deposit.

**Figure 3:**
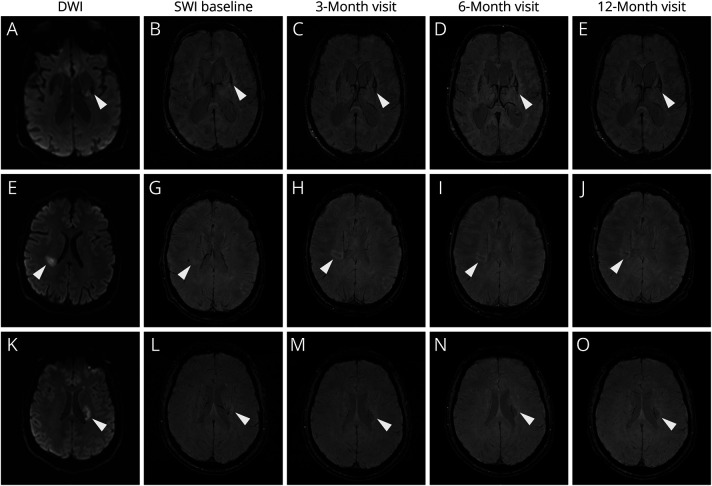
Morphology and Evolution of Hemosiderin Deposits (A–E) In a patient with an infarct in the basal ganglia, the “rim” pattern developed and became more obvious and thicker along the follow-ups. (F–J) In a patient with a RSSI adjacent to the lateral ventricle, the “smudge” became larger at 3-month and 6-month visits but shrank and turned darker, “condensed,” at the 12-month visit. (K–O) A “cluster” pattern in another RSSI adjacent to the lateral ventricle and the number of dots and lines became more visible inside and around the infarct during follow-up. DWI = diffusion-weighted imaging; RSSI = recent small subcortical infarct.

The categories and morphologies of HDs are illustrated in Figure 2 and given in eTable 1. HDs were observed in 43 (55.0%) of 108 within 6 months and 83 (76.9%) of 108 within 12 months of follow-up, with the mean time to first HD detection being 87 (IQR 53–164) days. Of the 83 participants who developed HDs, 43 (55.0%) had appeared by the baseline scan (1–3 months) and 82 (98.8%) by the 6-month follow-up scan (eTable 1), mainly due to the increase in “smudge,” “rim,” and “cluster” patterns (Figure 3). Among all the HD types, the predominant one was “smudge” from the baseline to the 12-month follow-up. The “smudge” could become smaller and darker, diminish over time (eFigure 2), or transform into another pattern during the 12-month follow-up (Figure 3). The “rim” pattern (i.e., similar to long-term sequelae of a primary hemorrhage) occurred in 26.5% of RSSIs by the 6-month visit and 31.7% by the 12-month visit, mainly in RSSIs in the lentiform/internal capsule region (50.0%) or thalamus (36.4%) (eTable 1). The “rim” could be partial or total and appeared approximately at mean 123 (SD 94) days after stroke onset, with 23.1% manifesting at 6 months or later. The “cluster” pattern appeared in 20.9% of RSSIs at baseline and gradually increased, accounting for 25.3% at 12-month follow-up (eTable 1), presenting as lines/dots within the infarct, primarily seen in RSSIs located in white matter regions (Figure 3, K–O). The “rim” and “cluster” tended to become more obvious over time. Three participants developed a mixed type of “rim” and “cluster.” The “spot” pattern was the rarest and most stable (Figures 2A and 3, F–J).

### Factors Influencing Occurrence of HDs

A binary logistic regression model (Table 2) was constructed to include RSSI volume; location; total SVD score; NIHSS score; and demographic variables including age, sex, stroke or TIA, hypertension, hyperlipidemia, diabetes, and smoking. RSSI volume (odds ratio [OR] 1.003, 95% CI 1.001–1.006 per cubic millimeter increase; *p* = 0.004) and total SVD score (OR 2.35, 95% CI 1.20–4.61, *p* = 0.01) independently predicted HD occurrence at 12 months, but not RSSI location or prescribed stroke prevention medication (antiplatelet vs anticoagulant therapy) (Table 2, model 1). In a second logistic regression model including individual SVD imaging markers, the number of lacunes showed an association with the occurrence of HDs (OR 1.60, 95% CI 1.13–2.26, *p* < 0.01). No statistically significant relationships were observed for Fazekas score, number of microbleeds, and basal ganglia mineral deposit score (Table 2, model 2). In addition, no significant association was found between WMH volume and HDs (eTable 2). No collinearity was observed among the individual SVD features (eTable 3). Regarding clinical outcomes, we did not find strong evidence of a link between HDs and mRS score ≥2 at the 12-month follow-up (Table 3), nor between HDs and MoCA scores at the 12-month follow-up (Table 4). Linear regression model assumptions were met as shown by the normality of residuals (eFigure 3). The logistic regression models showed good discrimination (area under the curve >0.88) and calibration (eFigure 4).

**Table 2:** Factors Predicting Hemosiderin Deposits: Adjusted Binary Logistic Regression Models

Variables	OR (95% CI)	*p* Value
Model 1		
Age	0.98 (0.92–1.04)	0.43
Sex	2.12 (0.60–7.47)	0.24
NIHSS score	0.87 (0.58–1.29)	0.48
Stroke or TIA history	0.33 (0.07–1.59)	0.17
Diabetes	0.36 (0.10–1.31)	0.12
Smoking	2.18 (0.68–6.99)	0.19
Therapy	0.36 (0.06–2.24)	0.28
Volume of index RSSIs, mm^3^	1.003 (1.001–1.006)	0.005
Location of hemosiderin deposits	0.71 (0.19–2.61)	0.60
Total SVD score at baseline	2.17 (1.13–4.18)	0.02
Model 2		
Age	1.03 (0.96–1.11)	0.36
Sex	2.42 (0.64–9.14)	0.19
NIHSS	0.78 (0.45–1.14)	0.27
Stroke or TIA history	0.22 (0.04–1.22)	0.08
Diabetes	0.29 (0.07–1.20)	0.09
Smoking	2.07 (0.60–7.20)	0.25
Therapy	0.30 (0.05–1.95)	0.21
Volume of index RSSIs, mm^3^	1.003 (1.001–1.006)	0.004
Location of hemosiderin deposits	0.73 (0.19–2.82)	0.65
Iron deposits in the basal ganglia	0.61 (0.28–1.34)	0.22
Fazekas score	0.90 (0.57–1.44)	0.67
No. of microbleeds	1.03 (0.90–1.18)	0.70
No. of lacunes	1.52 (1.07–2.16)	0.02

Abbreviations: NIHSS = NIH Stroke Scale; OR = odds ratio; RSSI = recent small subcortical infarct; SVD = small vessel disease; TIA = transient ischemic attack.

Receiver operating characteristic curves: model 1: the area under the curve was 0.87 with 95% CI 0.79–0.95 (*p* < 0.001); model 2: the area under the curve was 0.89 with 95% CI 0.82–0.96 (*p* < 0.001).

**Table 3:** Association Between Hemosiderin Deposits and mRS: Adjusted Binary Logistic Regression Model (N = 104, Smoking Status Missing = 3, mRS Score Missing = 1)

	mRS score ≥2	
	OR (95% CI)	*p* Value
Age	0.96 (0.90–1.03)	0.22
Sex	0.76 (0.18–3.17)	0.71
NIHSS	2.15 (1.35–3.43)	0.001
BMI	1.15 (1.00–1.33)	0.049
History of stroke or TIA	0.29 (0.04–1.93)	0.20
Diabetes	4.57 (0.98–21.34)	0.05
Hypertension	0.29 (0.05–1.77)	0.18
Hypercholesterolemia	1.27 (0.20–7.90)	0.80
Smoking	2.79 (0.66–11.76)	0.16
Hemosiderin deposits	0.21 (0.04–1.07)	0.06
Total SVD score at baseline	2.10 (1.09–4.07)	0.03

Abbreviations: BMI = body mass index; mRS = modified Rankin Scale; NIHSS = NIH Stroke Scale; OR = odds ratio; SVD = small vessel disease; TIA = transient ischemic attack.

Receiver operating characteristic curves: the area under the curve was 0.88 with 95% CI 0.79–0.96 (*p* < 0.001).

**Table 4:** Association Between Hemosiderin Deposits and MoCA: Multiple Linear Regression Model (N = 97, MoCA Missing = 11)

	Unstandardized B	Standardized coefficient beta	95% CI for unstandardized B	*p* Value
Age	−0.04	−0.15	−0.10 to 0.10	0.11
Sex	−0.52	−0.07	−1.81 to 0.77	0.42
BMI	−0.03	−0.04	−0.15 to 0.10	0.68
NIHSS	−0.98	−0.44	−1.37 to −0.58	<0.001
Stroke or TIA history	−1.72	−0.20	−3.33 to −0.12	0.04
Diabetes	−0.95	−0.12	−2.36 to 0.47	0.19
Hypertension	0.14	0.02	−1.27 to 1.55	0.84
Hypercholesterolemia	1.29	0.17	−0.08 to 2.67	0.07
Smoking	0.77	0.12	−0.36 to 1.89	0.18
Hemosiderin deposits	−0.33	−0.04	−1.77 to 1.10	0.64
Total SVD score at baseline	−0.10	−0.04	−0.61 to 0.40	0.68

Abbreviations: BMI = body mass index; MoCA = Montreal Cognitive Assessment; NIHSS = NIH Stroke Scale; OR = odds ratio; SVD = small vessel disease; TIA = transient ischemic attack.

The result of the linear regression indicated that the regression model is statistically significant in explaining the variance in the dependent variable (*F* = 4.756, *p* < 0.001). Assumptions of linear regression were checked graphically and were broadly met.

## Discussion

We describe the morphology and evolution of HDs as a long-term imaging marker of RSSIs on SWI sequences in 108 participants over the first year after clinically evident lacunar ischemic stroke. HDs were observed in 43 (55.0%) of 108 within 6 months and 83 (76.9%) of 108 within 12 months of stroke. Of the 4 different patterns of HDs identified, the initial and prevailing type was the “smudge” pattern (Figure 1) while the “rim” pattern that could be mistaken for primary hemorrhage was present in over 25% of RSSIs from 6-month follow-up onward. HDs were independently associated with larger RSSI volumes at the acute stage and with a higher total SVD score.

Our study may have important clinical implications. First, we describe and subcategorize in detail a distinctive image marker on SWI, HDs in RSSIs, providing additional insights beyond degrees of cavitation or disappearance of RSSI after lacunar ischemic stroke. The predominant “smudge” type persisted throughout the year of follow-up while “rim” and “cluster” patterns demonstrated characteristic distributions, primarily in basal ganglia gray matter and in white matter regions, respectively. Our study also indicated that the HD appearance changed over time. The smudge could become darker, diminish (eFigure 2), or evolve into a rim or cluster while “rim” and “cluster” patterns often combined with “smudge.” There were also a few cases developing into the “rim and cluster mixed” type. The rare “spot” pattern remained stable over time, emphasizing the stability of this particular HD type.

Second, the identification of the “rim” pattern in over 25% of SSIs at all follow-up time points holds clinical importance and research interest. Some hemosiderin rims were striking, potentially leading to misinterpretation as sequelae of a primary hemorrhage rather than an infarct had the initial diagnostic imaging not shown that the lesion was ischemic. Contrary to previous studies suggesting hemorrhagic foci as indicative of hemorrhagic transformation in high-risk populations,^7^ our findings suggest that the “rim” pattern could evolve from the “smudge” pattern over time. It is important to note that HDs, including the “rim” pattern, were not associated with the number of microbleeds, other types of bleeding, or prescribed antiplatelet or anticoagulant therapy in adjusted analyses, reducing the likelihood of attributing HD patterns solely to hemorrhagic transformation due to antiplatelet or anticoagulant treatment. The “rim” pattern typically was detected approximately at mean 123 (SD 94) days after stroke onset, with 23.1% manifesting at 6 months or later, indicating its association with chronic, slowly evolving, pathologic changes in RSSIs. Therefore, the presence of HDs should not be a contraindication for antithrombotic therapy.

Third, different HD patterns may stem from distinct mechanisms. Given the heterogeneity of SVD involving arterioles, capillaries, and venules,^1^ SWI findings could reflect red blood cell leakage from vessels due to blood-brain barrier damage, leading to hemosiderin visibility and accumulation.^15-18^ This might explain why the “smudge” predominated among the RSSIs. Possibly, it might indicate neovascularization into the damaged tissue, with newly forming vessels typically being leaky leading to HDs. Moreover, the location of infarcts might determine HD categorization, with “rim” patterns more common in lenticulostriate artery (LSA) territories such as the basal ganglia and thalamus while “cluster” patterns prevail in white matter regions such as the corona radiata and centrum semiovale. This might reflect a similar process to the vessel clusters seen in WMH tending to cavitate,^19^ possibly to arteriole to venule shunting,^20^ venous collagenosis,^21,22^ or arteriolar tortuosity.^23^

Finally, infarct volume, total SVD score, and number of lacunes were identified as potential predictive image markers of HDs, highlighting the significance of the initial infarct severity and chronic SVD pathology. Our findings align with previous studies, suggesting that HDs may be widespread but underrecognized in participants with SVD. Among participants with noncardioembolic stroke or TIA, 10.4% showed hemorrhagic dark signal at the RSSI lesion on follow-up. The large lesion size and anterior circulation location were associated with hemorrhagic foci, rather than the number of CMBs, history of intracranial hemorrhage, cavitary change, and antiplatelet type.^7^ Furthermore, another study revealed that 44.6% of RSSIs in the territory of LSA (the basal ganglia) exhibited hemorrhagic foci. These foci were linked to larger lesion volumes, increased cavitation formation, shorter total LSA length, higher baseline NIHSS scores, and poorer functional outcomes.^8^ Of interest, a vessel cluster sign was observed in the white matter region of 33.33% of participants with sporadic SVD. This sign correlated with more lacunes, higher WMH volume, and lower cerebrovascular reactivity in normal-appearing white matter.^21^ Our findings supported that the size of infarcts and the number of lacunes had an impact on HDs. In our study, each additional cubic millimeter increase in infarct volume was associated with a 0.3% increase in the odds of having HDs and no specific cutoff value was identified. However, divergent results were observed for WMH volume, treatment, and clinical outcomes. This inconsistency could be attributed to differences in the study population and sample size. One strength of our study was the more frequent follow-up scanning, allowing us to track the formation and evolution of HDs more comprehensively than previous studies. Given the varied pathologies associated with different HD patterns, further longitudinal studies are warranted to explore the intricate relationship between HDs and SVD image markers, as well as clinical outcomes.

Our study has limitations. Owing to varying frequencies and time intervals of follow-up scans among participants in our study, the mean time from stroke onset to the first detection of HDs may not accurately reflect the duration of HD formation. In addition, further investigation into the relationship between HDs and therapy is warranted, particularly considering the absence of patients receiving thrombolysis in our study. While the individual subgroups are relatively small, the overall study encompasses a large sample size. Future studies could benefit from expansion to examine the frequency, location, influencing factors, and the association with clinical outcomes in larger cohorts. Moreover, caution is advised when identifying and interpreting HDs in clinical practice because distinguishing them from primary hemorrhage can be challenging without imaging from the acute stage and periodic follow-up. Our study is exploratory; hence, studies with larger sample sizes and longer term follow-ups are warranted to better characterize the evolution of HDs and the relationship with patient characteristics. The pathophysiologic significance of this new feature should also be confirmed through pathology studies and higher resolution imaging, such as 7T MRI.

In conclusion, HDs may occur in up to three-quarters of participants with RSSIs, and their presence on SWI may be associated with RSSI volume and the total SVD score. Caution is advised when identifying HDs to distinguish them from primary hemorrhage, particularly the “rim” pattern. This new imaging marker warrants further confirmation in future research to assess its evolution and contribution to SVD burden.

## Appendix 1 Authors

**Table:**

Name	Location	Contribution
Yu-Yuan Xu, MD	China National Clinical Research Center for Neurological Diseases, Beijing Tiantan Hospital, Capital Medical University	Drafting/revision of the manuscript for content, including medical writing for content; study concept or design; analysis or interpretation of data
Francesca M. Chappell, PhD	Centre for Clinical Brain Sciences, University of Edinburgh, United Kingdom	Drafting/revision of the manuscript for content, including medical writing for content; analysis or interpretation of data
Maria Del C. Valdés Hernández, PhD	Centre for Clinical Brain Sciences, UK Dementia Research Institute, University of Edinburgh, United Kingdom	Drafting/revision of the manuscript for content, including medical writing for content; major role in the acquisition of data
Carmen Arteaga-Reyes, PhD	Centre for Clinical Brain Sciences, UK Dementia Research Institute, University of Edinburgh, United Kingdom	Drafting/revision of the manuscript for content, including medical writing for content; major role in the acquisition of data
Una Clancy, PhD	Centre for Clinical Brain Sciences, UK Dementia Research Institute, University of Edinburgh, United Kingdom	Drafting/revision of the manuscript for content, including medical writing for content; major role in the acquisition of data
Daniela Jaime Garcia, PhD	Centre for Clinical Brain Sciences, UK Dementia Research Institute, University of Edinburgh, United Kingdom	Drafting/revision of the manuscript for content, including medical writing for content; major role in the acquisition of data
Stewart Wiseman, PhD	Centre for Clinical Brain Sciences, UK Dementia Research Institute, University of Edinburgh, United Kingdom	Drafting/revision of the manuscript for content, including medical writing for content; major role in the acquisition of data
Michael S. Stringer, PhD	Centre for Clinical Brain Sciences, UK Dementia Research Institute, University of Edinburgh, United Kingdom	Drafting/revision of the manuscript for content, including medical writing for content; major role in the acquisition of data
Michael Thrippleton, PhD	Centre for Clinical Brain Sciences, UK Dementia Research Institute, University of Edinburgh, United Kingdom	Drafting/revision of the manuscript for content, including medical writing for content; major role in the acquisition of data
Yajun Cheng, PhD	Department of Neurology, West China Hospital, Sichuan University, Chengdu, China	Drafting/revision of the manuscript for content, including medical writing for content; major role in the acquisition of data
Junfang Zhang, PhD	Department of Neurology & Institute of Neurology, Ruijin Hospital affiliated with Shanghai Jiao Tong University School of Medicine, China	Drafting/revision of the manuscript for content, including medical writing for content; major role in the acquisition of data
Xiaodi Liu, PhD	Division of Neurology, Department of Medicine, LKS Faculty of Medicine, The University of Hong Kong, China	Drafting/revision of the manuscript for content, including medical writing for content; major role in the acquisition of data
Angela C.C. Jochems, PhD	Centre for Clinical Brain Sciences, UK Dementia Research Institute, University of Edinburgh, United Kingdom	Drafting/revision of the manuscript for content, including medical writing for content; major role in the acquisition of data
Fergus Doubal, MBChB, PhD	Centre for Clinical Brain Sciences, UK Dementia Research Institute, University of Edinburgh, United Kingdom	Drafting/revision of the manuscript for content, including medical writing for content; major role in the acquisition of data
Joanna M. Wardlaw, MD, FRCR, FMedSci	Centre for Clinical Brain Sciences, UK Dementia Research Institute, University of Edinburgh, United Kingdom	Drafting/revision of the manuscript for content, including medical writing for content; major role in the acquisition of data; study concept or design; analysis or interpretation of data

## Appendix 2 Coinvestigators

**Table:**

Name	Location	Role	Contribution
Ian Marshall, PhD	University of Edinburgh, United Kingdom	Emeritus Chair of Magnetic Resonance Physics	Medical physics
Susana Muñoz Maniega, PhD	Centre for Clinical Brain Sciences, University of Edinburgh, United Kingdom	Research Fellow	Image analysis
Eleni Sakka, PhD	Centre for Clinical Brain Sciences, University of Edinburgh, United Kingdom	Research Fellow	Image analysis
Rosalind Brown, PhD	Centre for Clinical Brain Sciences, UK Dementia Research Institute, University of Edinburgh, United Kingdom	Research Manager	Study administration
Olivia K.L. Hamilton, PhD	Centre for Clinical Brain Sciences, UK Dementia Research Institute, University of Edinburgh, United Kingdom	Research Fellow	Data acquisition
Ellen Backhouse, PhD	Centre for Clinical Brain Sciences, UK Dementia Research Institute, University of Edinburgh, United Kingdom	Research Fellow	Data acquisition and analysis
Will Hewins, BSc, MSc	Centre for Clinical Brain Sciences, UK Dementia Research Institute, University of Edinburgh, United Kingdom	Assistant Psychologist	Study co-ordination; data acquisition
Rachel Locherty	Centre for Clinical Brain Sciences, UK Dementia Research Institute, University of Edinburgh, United Kingdom	Research Nurse	Study co-ordination; data acquisition
Emilie Sleight, PhD	Centre for Clinical Brain Sciences, UK Dementia Research Institute, University of Edinburgh, United Kingdom	PhD Student (VRS)	MRI vascular function acquisition/analysis
Alasdair G. Morgan, MD	Centre for Clinical Brain Sciences, UK Dementia Research Institute, University of Edinburgh, United Kingdom	PhD Student (VRS)	MRI vascular function acquisition/analysis
Iona Hamilton	Edinburgh Imaging Facility (Royal Infirmary of Edinburgh), University of Edinburgh, United Kingdom	Senior Radiographer	Radiographers
Gayle Barclay	Edinburgh Imaging Facility (Royal Infirmary of Edinburgh), University of Edinburgh, United Kingdom	Senior Research MRI Radiographer	Radiographers
Donna McIntyre	Edinburgh Imaging Facility (Royal Infirmary of Edinburgh), University of Edinburgh, United Kingdom	MRI Research Radiographer	Radiographers
Charlotte Jardine	Edinburgh Imaging Facility (Royal Infirmary of Edinburgh), University of Edinburgh, United Kingdom	MRI Research Radiographer	Radiographers
Dominic Job, PhD	University of Edinburgh, United Kingdom	Senior Research Data Manager	Programming/data management
David Perry, PhD	University of Edinburgh, United Kingdom	Research Technology Manager	Programming/data management
Tom MacGillivray, PgCAP	Centre for Clinical Brain Sciences, University of Edinburgh, United Kingdom	Senior Research Fellow & Image Analysis Core Laboratory Manager	Retinal studies
Charlene Hamid	University of Edinburgh, United Kingdom	Lead Ophthalmic Imager and Analysist	Retinal data acquisition and analysis
Salvatore Rudilosso, PhD, MB ChB	Comprehensive Stroke Center, Department of Neuroscience, Hospital Clinic, Barcelona, Spain	Visiting Neurology Fellow	DTI+ve lesion analysis

## Glossary

HD: hemosiderin deposit
IQR: interquartile range
LSA: lenticulostriate artery
MoCA: Montreal Cognitive Assessment
mRS: modified Rankin Scale
MSS3: Mild Stroke Study 3
NIHSS: NIH Stroke Scale
OR: odds ratio
RSSI: recent small subcortical infarct
SVD: small vessel disease
SWI: susceptibility-weighted imaging
TIA: transient ischemic attack
WMH: white matter hyperintensity
